# Supplementary material for: Bridging multiscale interfaces for developing ionically conductive high-voltage iron sulfate-containing sodium-based battery positive electrodes
Source: Nat Commun. 2023 Jun 22;14:3701. doi: 10.1038/s41467-023-39384-7 (PMC10287750; doi:10.1038/s41467-023-39384-7)
Supplement: Supplementary file 1 — Supplementary Information [file 41467_2023_39384_MOESM1_ESM.pdf]

Supplementary Information

Bridging multiscale interfaces for developing ionically conductive high-voltage iron sulfate-containing sodium-based battery positive electrodes

Jiyu Zhang,[a] Yongliang Yan,[a] Xin Wang,[a] Yanyan Cui,[b] Zhengfeng Zhang,[c] Sen Wang,[a] Zhengkun Xie,[a] Pengfei Yan,[c] Weihua Chen[a],*

[a] College of Chemistry & Green Catalysis Center, Zhengzhou University, Zhengzhou 450001, Henan, China

[b] Institute of Nanotechnology, Karlsruhe Institute of Technology (KIT), Hermann-von-Helmholtz-Platz 1, 76344 Eggenstein Leopoldshafen, Germany

[c] Beijing Key Laboratory of Microstructure and Properties of Solids, Faculty of Materials and Manufacturing, Beijing University of Technology, Beijing 100124, China

*E-mail: chenweih@zzu.edu.cn

J. Zhang and Y. Yan contributed equally to this work

Supplementary Figures

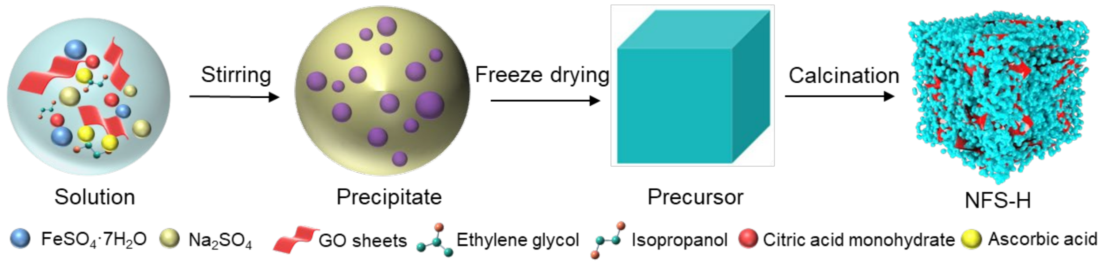

Supplementary Figure 1 | Schematic synthesis process of NFS-H. Within the solution process, graphene oxide (GO) sheets with electron-rich functional groups were added to provide adequate adsorption sites for positive charged Fe^{2+} and Na^{+}. During the subsequent co-precipitation process, excess instilled isopropyl alcohol acts as a precipitator to control the formation rate and nucleation size of nano-sized precursor precipitate, and a viscous glycol-containing solution environment enables a spontaneous assembly of precipitate without excessive agglomeration.

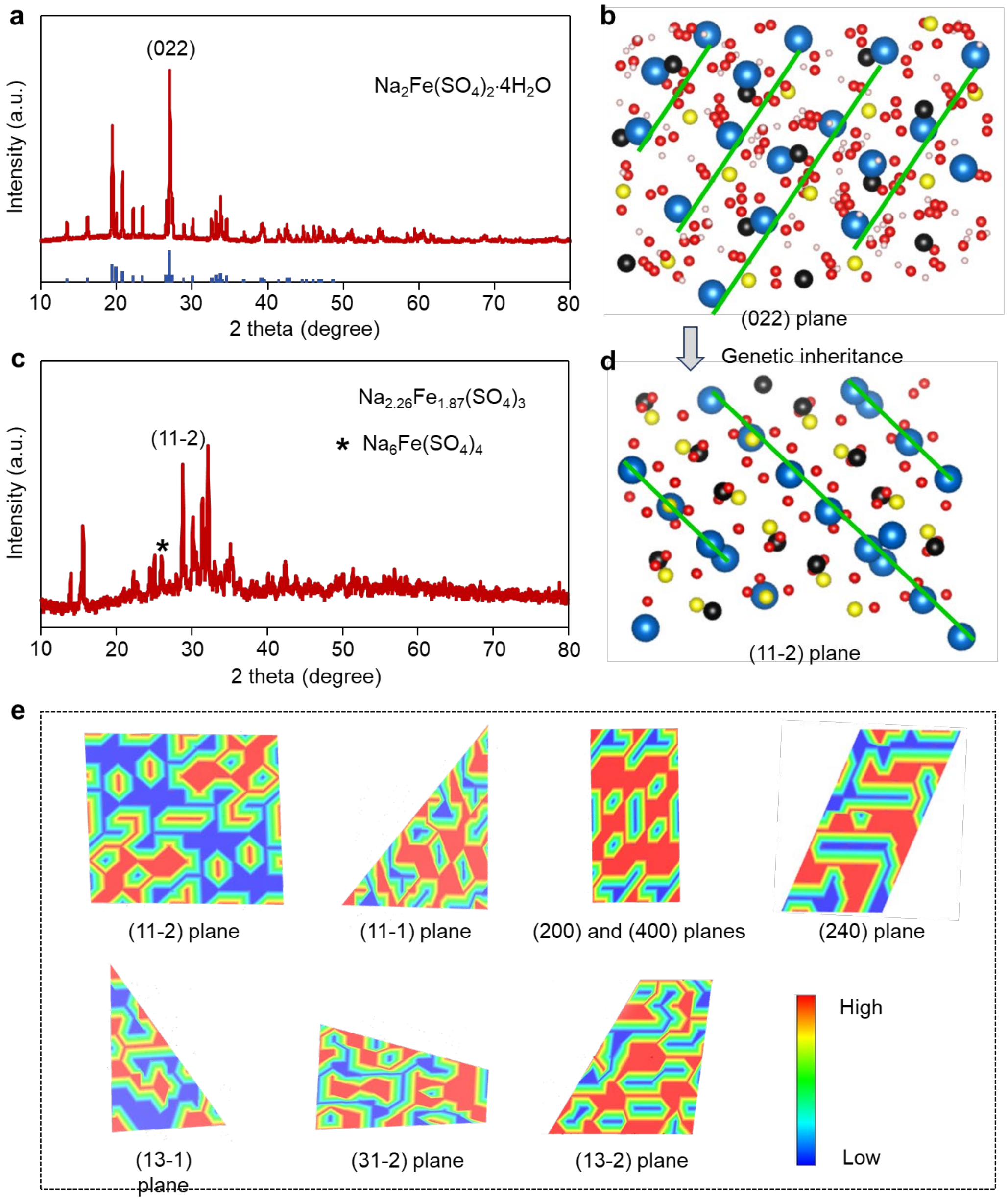

Supplementary Figure 2 | XRD patterns and crystallographic plane structure of NFS-H. a XRD patterns of precursor. **b** The crystallographic structure of (022) plane in $Na_2Fe(SO_4)_2{\cdot}4H_2O$ crystal. Na (blue-green), Fe (dark), S (buff), O (red) and H (light pink). **c** X-ray diffraction Rietveld refinement of NFS-H. Detailed crystallographic parameters are listed in Supplementary Tables 3 and 4. **d** The crystallographic structure of (11-2) plane in $Na_{2.26}Fe_{1.87}(SO_4)_3$ crystal. **e** Calculated electron density distributions of various crystal planes in $Na_{2.26}Fe_{1.87}(SO_4)_3$ crystals.

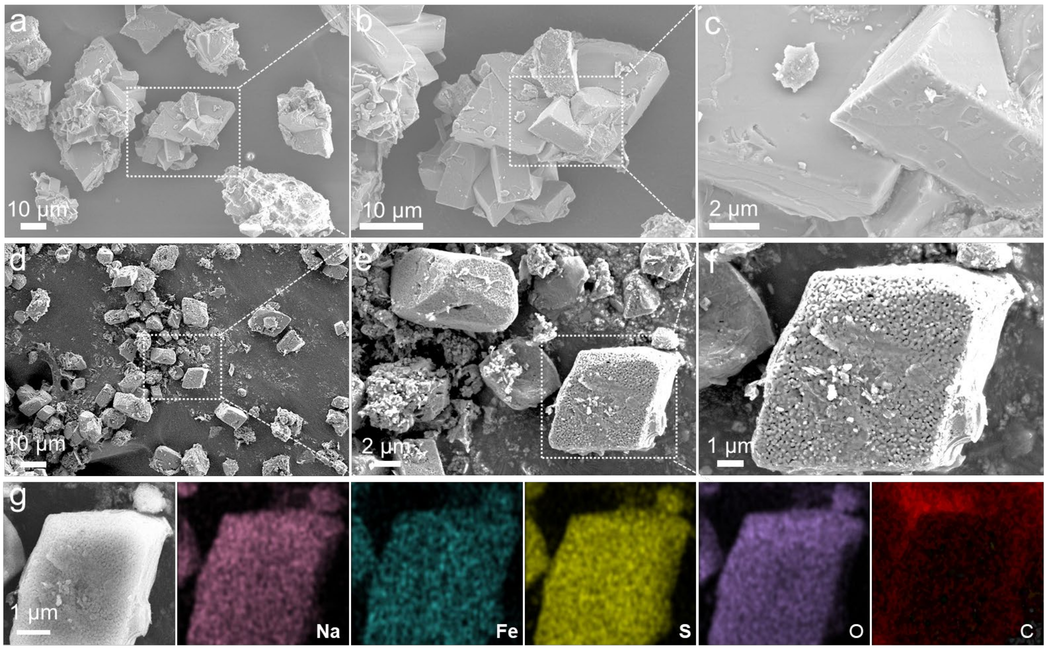

Supplementary Figure 3 | SEM images. a–c precursor and **d–f** NFS-H. **g** EDS mapping images of NFS-H. SEM images of NFS-H feature micron-sized polyhedron consisting of plenty of stacked nanoparticles. This rhomb-like morphology and size are inherited from the precursor with a smooth surface, except for the rough surface due to the carbonization and dehydration of precursor.

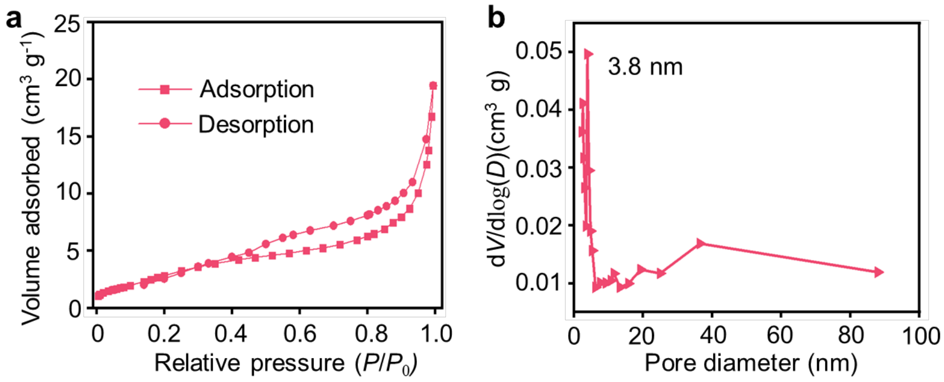

Supplementary Figure 4 | Characterization of the NFS-H porosity. a N_2 adsorption-desorption and **b** corresponding pore size distribution. N_2 adsorption–desorption measurement of NFS-H implies an II-type isotherm with a distinguishable capillary condensation step and a Brunauer-Emmett-Teller (BET) surface area (8.15 m^2 g^{-1}). The typical H3-type hysteresis loop indicates a slit pore structure with a size varying from 2 to 10 nm.

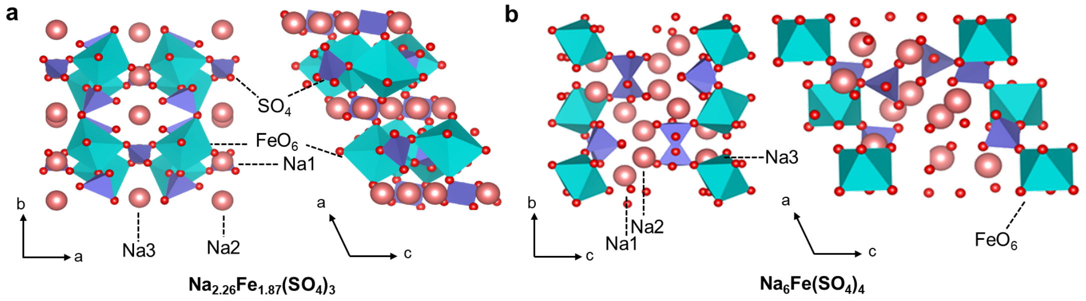

Supplementary Figure 5 | Crystal structures. a $Na_{2.26}Fe_{1.87}(SO_4)_3$. **b** $Na_6Fe(SO_4)_4$. Pink and red balls represent Na and O atoms, respectively. Green octahedrons and blue tetrahedron represent FeO_6 octahedra and SO_4 tetrahedra units, respectively.

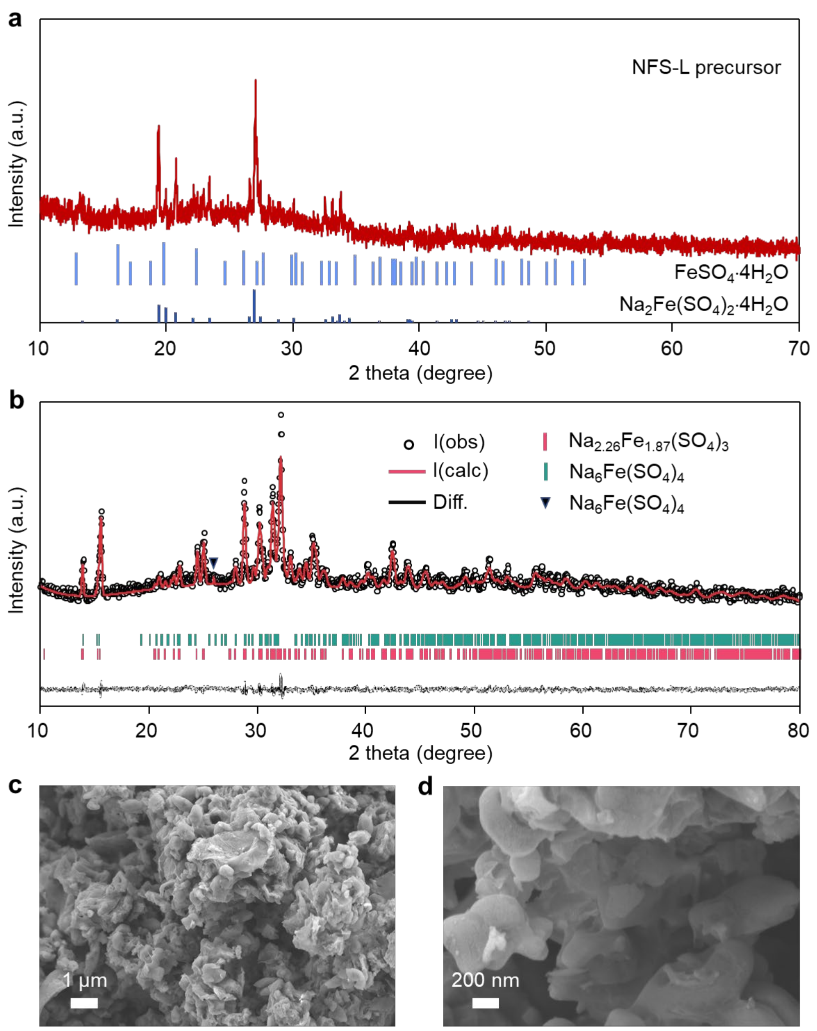

Supplementary Figure 6 | Structure and morphology characterizations of NFS-L. a XRD pattern of precursor. **b** X-ray diffraction Rietveld refinement and **c, d** SEM images. The precursor of NFS-L shows additional $FeSO_4$ $4H_2O$ peaks, indicating that the ethylene glycol (EG)-free precursor solution facilitates single-phase precipitated $FeSO_4·4H_2O$ in precursor. The peak at 26.05° corresponding to $Na_6Fe(SO_4)_4$ shows a weaker intensity compared to that in NFS-H (Supplementary Fig. 2), implying the less content of $Na_6Fe(SO_4)_4$ phase. These result reveals that, excess $FeSO_4$ components might help to reduce the formation of Na-rich $Na_6Fe(SO_4)_4$ phase in products.

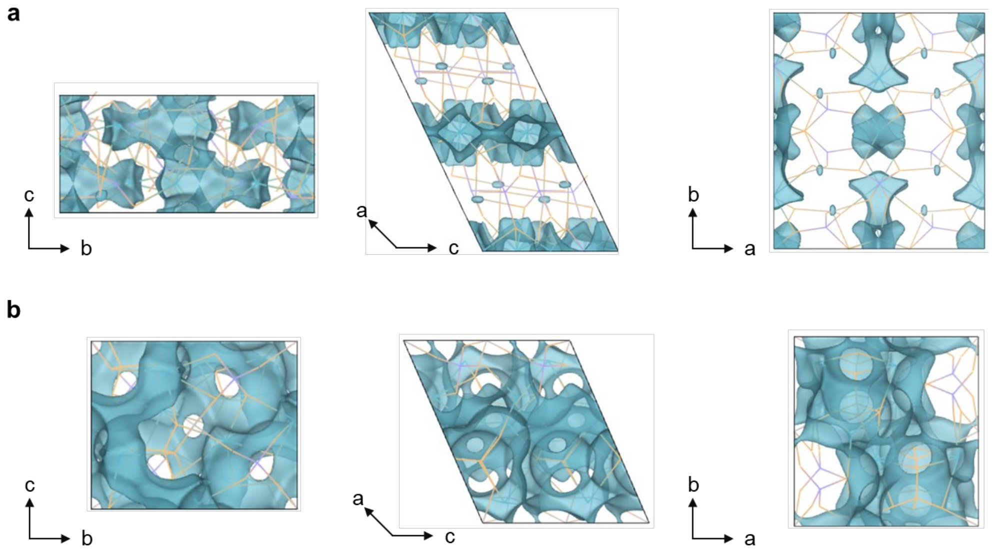

Supplementary Figure 7 | The Na^+ migration channels (blue-green isosurface). a $Na_{2.26}Fe_{1.87}(SO_4)_3$ crystal. **b** $Na_6Fe(SO_4)_4$ crystal. The migration pathways of mobile Na^+ were calculated based on the bond-valence site energy (BVSE) method and Voronoi decomposition (CAVD), which was carried out on Computing and Date Platform for Electrochemical Energy Storage Materials.

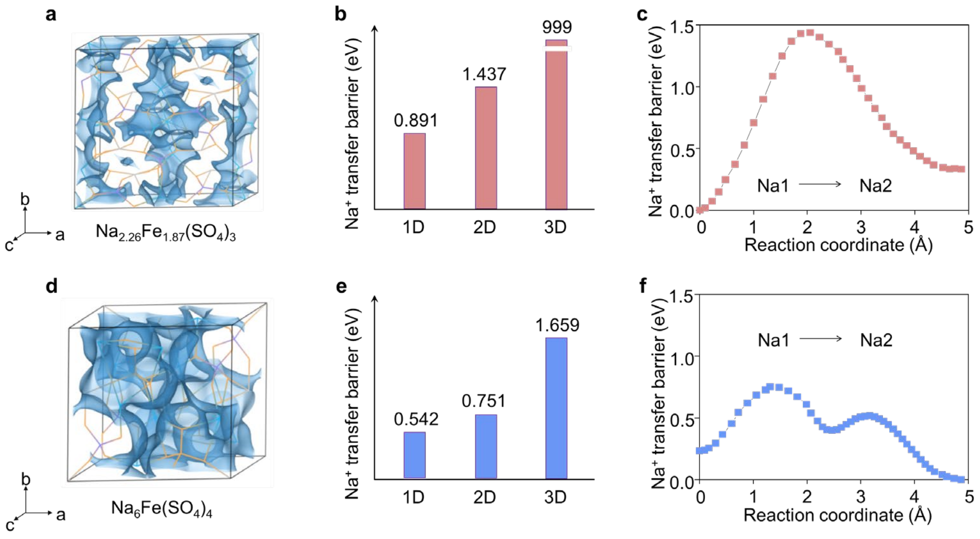

Supplementary Figure 8 | Na^+ migration channels and energy barriers revealed by BVSE calculation. Na^+ migration channels barriers (blue-green isosurface) along different directions and energy profiles of path Na1–Na2 for **a–c** $Na_{2.26}Fe_{1.87}(SO_4)_3$ and **d–f** $Na_6Fe(SO_4)_4$ crystals, respectively. The BVSE calculation was performed on SoftBV software, and the resolution is 0.1. Related discussion is presented in Supplementary Note 1.

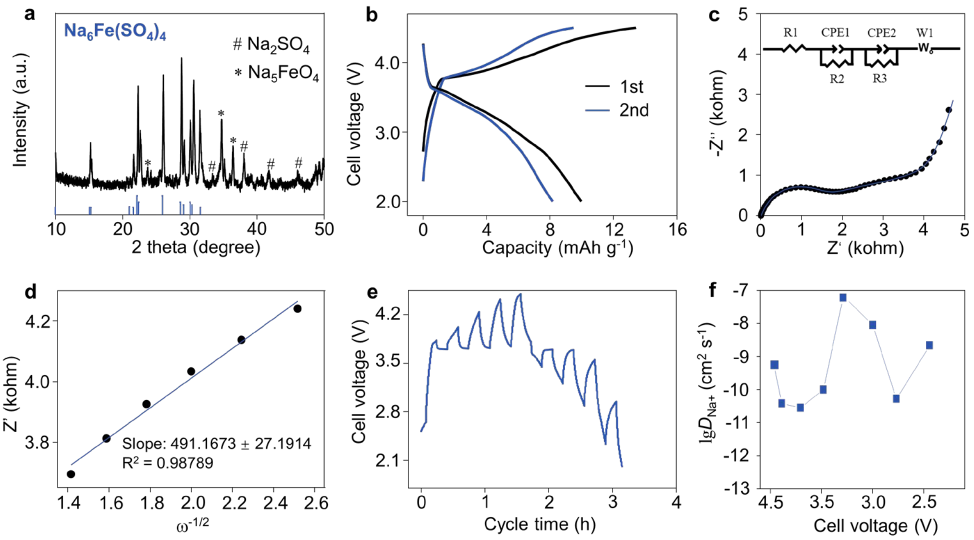

Supplementary Figure 9 | Structural and electrochemical characterization of $Na_6Fe(SO_4)_4$. **a** XRD pattern. **b** Charge/discharge potential profiles of the Na||$Na_6Fe(SO_4)_4$ coin cell at 12 mA g^{-1}. **c** EIS plot of prepared $Na_6Fe(SO_4)_4$ electrode after initial cycle (line: fitted data; dot: pristine data). In the fitted equivalent circuit, R1 represents the ohmic resistance in the cell system. Meanwhile, R2 and CPE1 (constant phase angle element) correspond to the resistance and capacitance of interfacial CEI. In addition, R3, CPE2 and W1 represent the faradaic impedance of the reaction, the interphase double layer capacitance and the diffusion impedance, respectively. **d** Fitted Z'–$\omega^{-1/2}$ curve with its slope and error. Error bars represent standard deviations for fitted curve. **e** GITT curves. **f** Calculated Na^+ diffusion coefficients. Related discussion is presented in Supplementary Note 2.

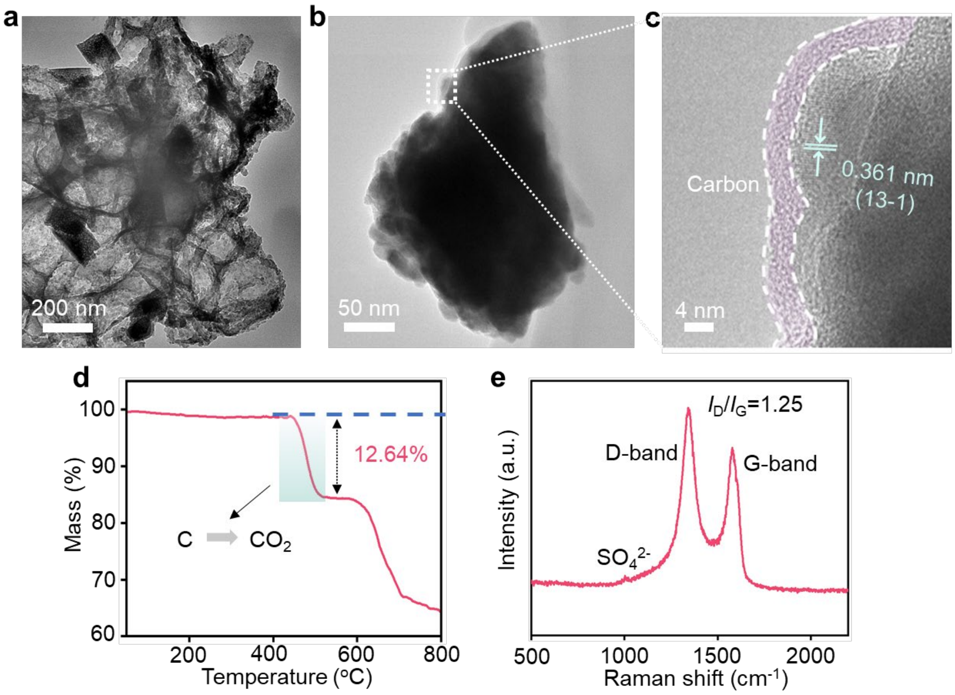

Supplementary Figure 10 | Morphological and physical characterizations of NFS-H. a–c TEM images of NFS-H material after ultrasonic treatment. **d** TG curve of NFS-H in air. **e** Raman spectrum.

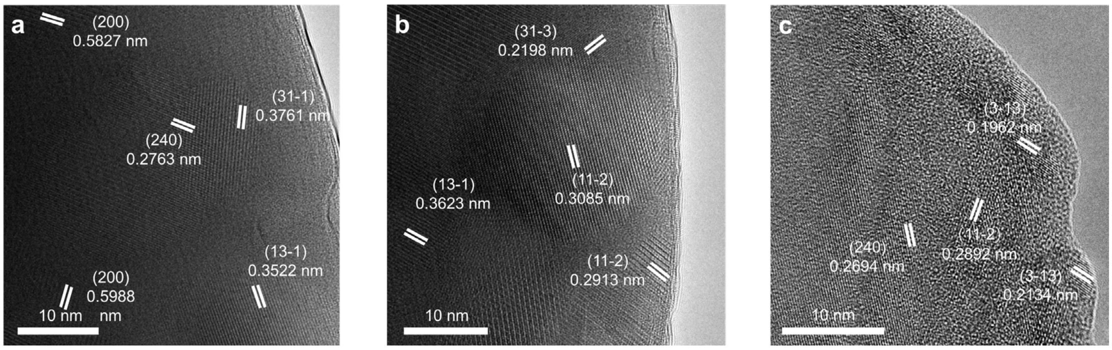

Supplementary Figure 11 | HAADF-STEM images of NFS-H at different regions. a Region I. **b** Region II. **c** Region III. The particles investigated were powder. Related discussion is presented in Supplementary Note 3.

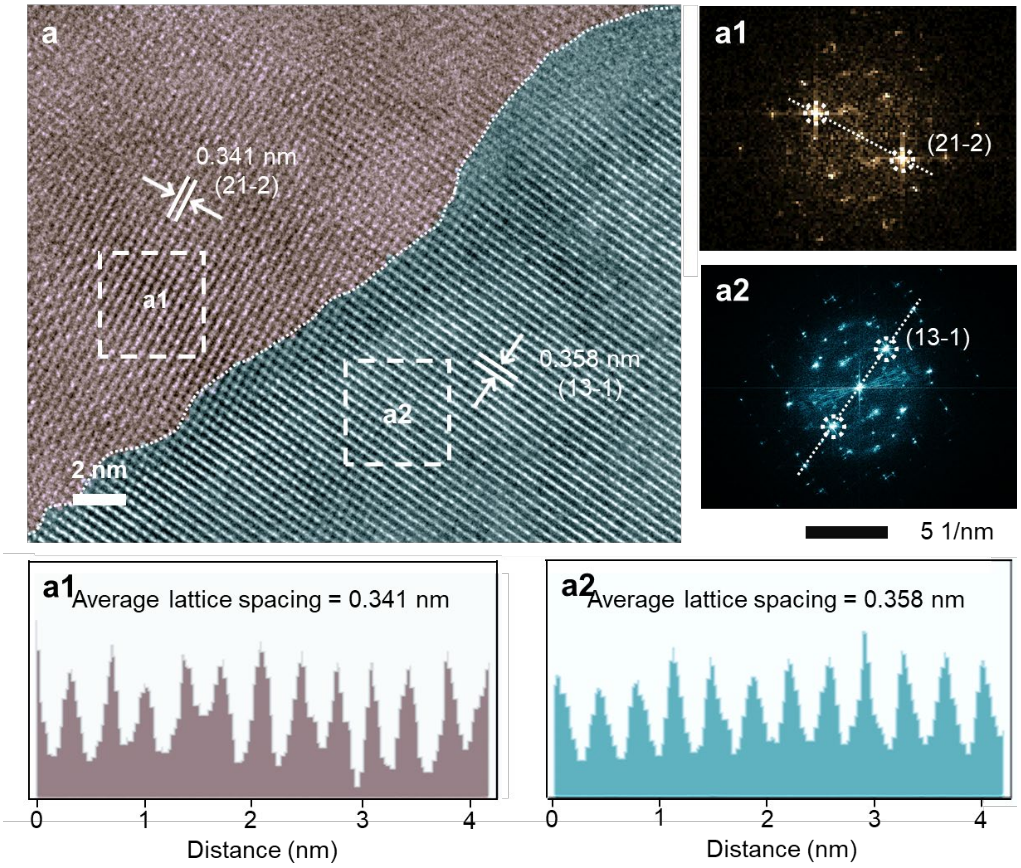

Supplementary Figure 12 | HAADF-STEM images of NFS-H material with corresponding line scans and FFT patterns. The particles investigated were powder. The light brown and light blue regions stand for the $Na_6Fe(SO_4)_4$ and $Na_{2.26}Fe_{1.87}(SO_4)_3$ crystals, respectively. The a1 and a2 indicates the square regions in figure **a**.

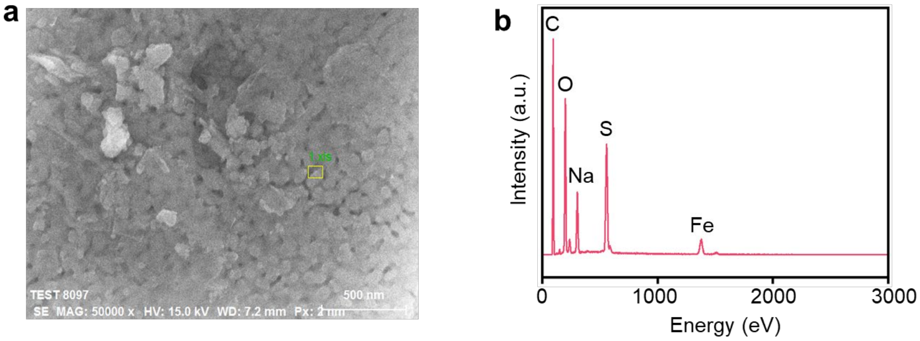

c

El	AN	Series	unn. [wt.%]	C norm. [wt.%]	C Atom. [at.%]	C Error (1 Sigma) [wt.%]
O	8	K-series	47.06	45.30	60.87	5.72
Fe	26	K-series	21.16	20.37	7.84	0.69
S	16	K-series	18.53	17.84	11.96	0.69
Na	11	K-series	12.39	11.93	11.15	0.81
C	6	K-series	4.75	4.57	8.18	1.11
		Total:	103.89	100.00	100.00	

Supplementary Figure 13 | EDS characterization of NFS-H. a Selected area. **b** EDS spectrum. **c** Element contents. Error represents standard deviations for detected element content.

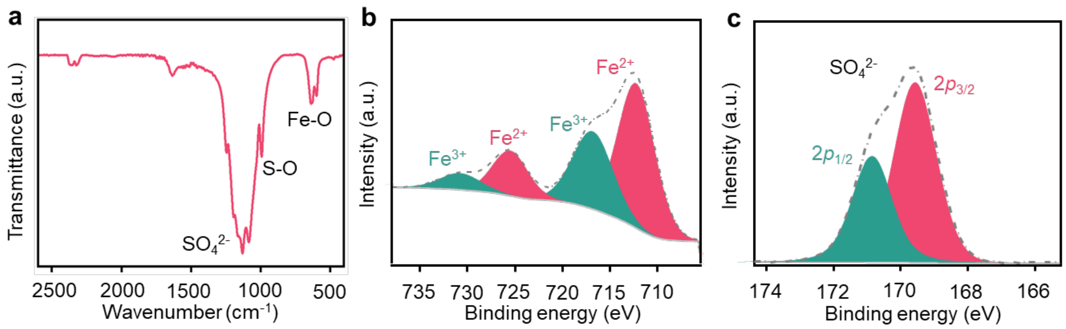

Supplementary Figure 14 | Spectroscopic characterizations of NFS-H. a FTIR, high-resolution **b** Fe 2*p* and **c** S 2*p* spectra. FT-IR spectrum shows the characteristic peaks of SO_4^{2-} group (at 622, 989, and 1078 cm^{-1}) and the vibrational bonds between Fe^{2+} and O^{2-} in the isolated FeO_6 octahedra at 601 cm^{-1}. Moreover, high-resolution XPS spectra (Fe 2*p* and S 2*p*) present strong signals associated with Fe^{2+} and SO_4^{2-}, and a part of oxidized Fe^{3+} in the air.

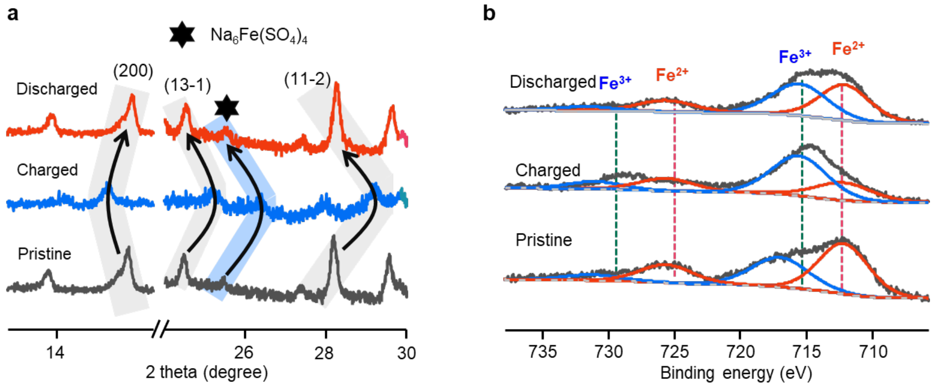

Supplementary Figure 15 | The characterizations on reaction mechanism of NFS-H. Ex situ **a** XRD patterns and **b** XPS spectra of NFS-H cathode during initial charge-discharge process. The Na||NFS-H coin cells were stopped and disassembled upon initial cycles at various state (Open circuit voltage, charge at 4.5 V, and discharge at 2.0 V) to conduct the XRD and XPS tests. Storage sodium mechanism investigation of NFS-H cathode is actualized via ex situ XRD patterns and XPS spectra upon the initial cycle. At charged 4.5 V state, the peaks at 15.5°, 28.8° and 30.1° show slight shift to lower angles due to the desodiation of lattice structures. Meanwhile, due to Na^+ migration at Na2 and Na3 sites along the *c* axis, the peak intensities at 31.3° and 32.0° weaken. When backed to 2.0 V, the diffraction peaks reappear or return to original angles, indicating the reversible phase transformation. XPS spectra show similar change of Fe valance, supporting the solid-solution transition mechanism of NFS-H cathode during electrochemical process.

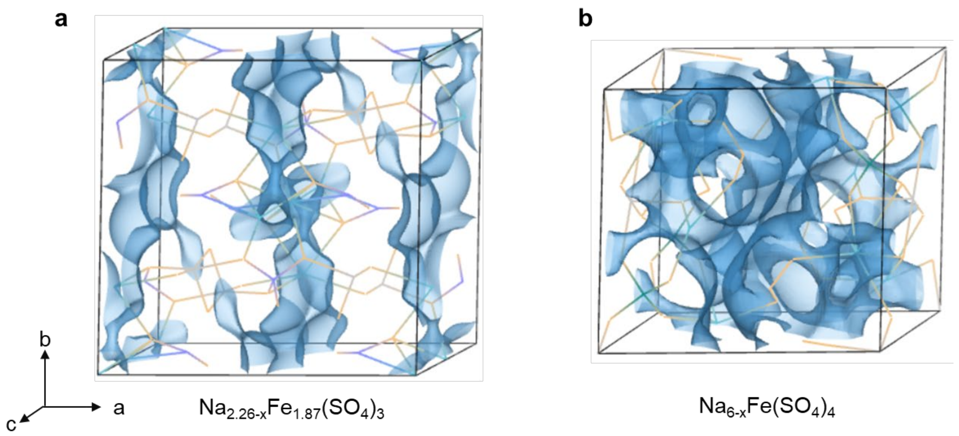

Supplementary Figure 16 | Na$^+$ migration channels (blue-green isosurface) revealed by BVSE calculation. **a** $Na_{2.26-x}Fe_{1.87}(SO_4)_3$. **b** $Na_{6-x}Fe(SO_4)_4$. The calculation was performed on SoftBV software, and the resolution is 0.1.

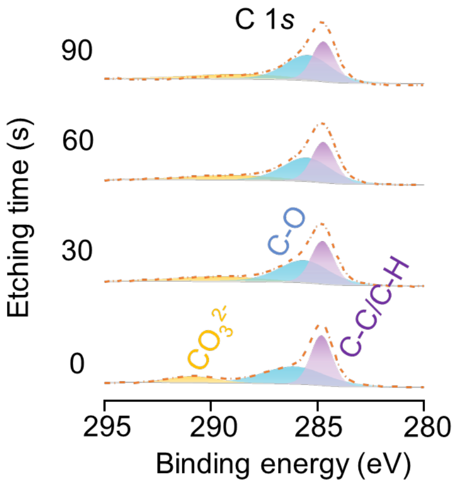

Supplementary Figure 17 | In-depth element distributions and high-resolution ex situ XPS spectra of C 1*s* on NFS-H electrode at charged 4.5 V upon initial cycle.

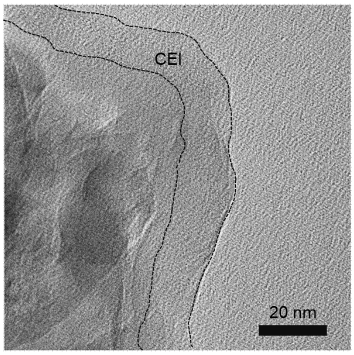

Supplementary Figure 18 | Ex situ cryo-TEM image of NFS-H cathode after 70 cycles, showing the presence of a CEI layer with a thickness of 12−17 nm.

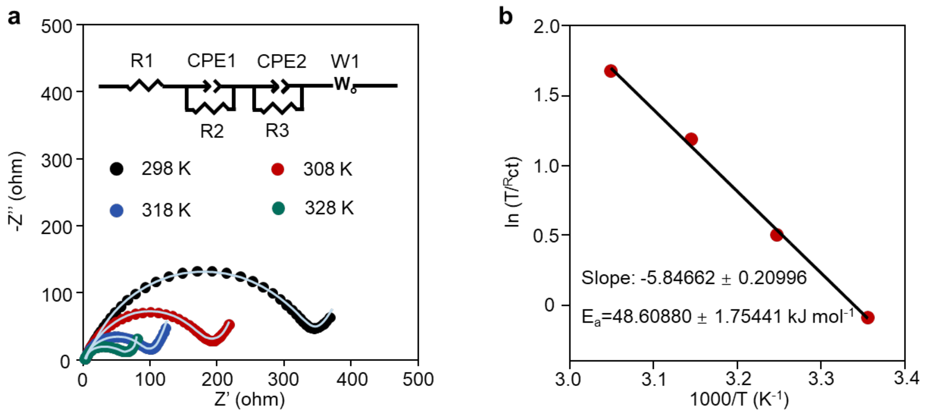

Supplementary Figure 19 | Electrochemical impedance of Na||NFS-H cells after initial cycle. a EIS plots at different temperatures (line: fitted data; dot: pristine data). In the fitted equivalent circuit, R1 represents the ohmic resistance in the cell system. Meanwhile, R2 and CPE1 (constant phase angle element) correspond to the resistance and capacitance of interfacial CEI. In addition, R3, CPE2 and W1 represent the faradaic impedance of the reaction, the interphase double layer capacitance and the diffusion impedance, respectively. Detailed resistance values are listed in Supplementary table 5. **b** The calculated activation energies of Na^{+} transfer across the interface with the error. Error bars represent standard deviations for fitted curve.

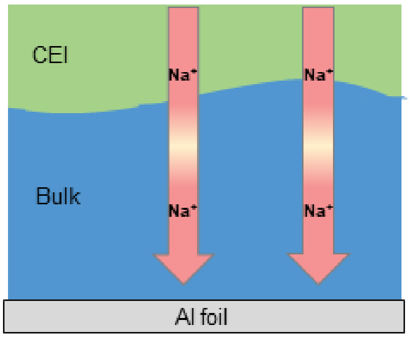

Supplementary Figure 20 | Scheme of designed continuous Na^{+} channels in cathodes.

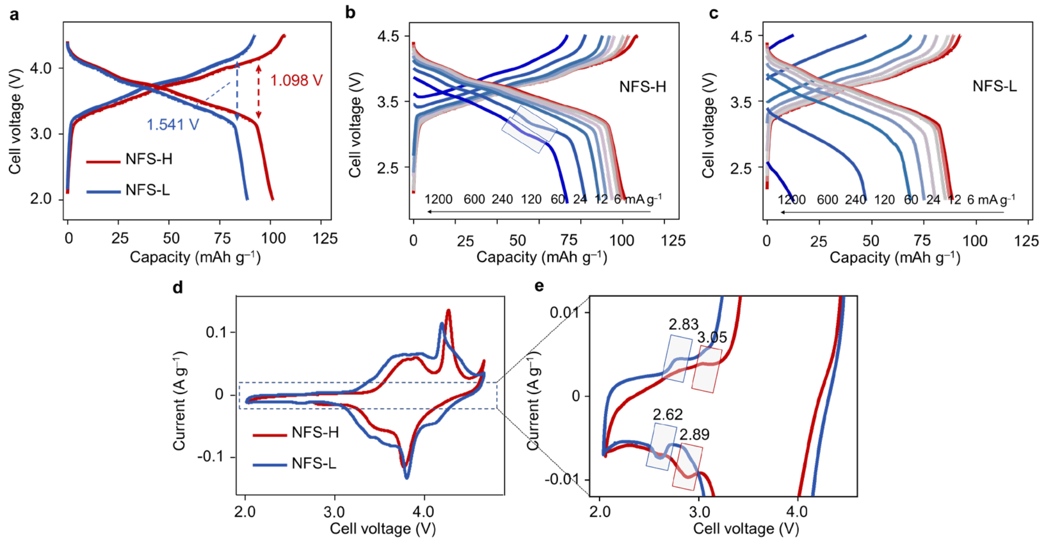

Supplementary Figure 21 | Electrochemical characterizations of NFS-H- and NFS-L-based electrodes in Na metal coin cell configuration. a 3th cycle charge/discharge voltage profiles for Na||NFS-H and Na||NFS-L coin cells at 6 mA g^{-1}. **b** Rate performance of Na||NFS-H coin cells. **c** Rate performance of Na||NFS-L coin cells. **d** CV curves of Na||NFS-H and Na||NFS-L coin cells. **e** Enlarged CV curves. Related discussion is presented in Supplementary Note 4.

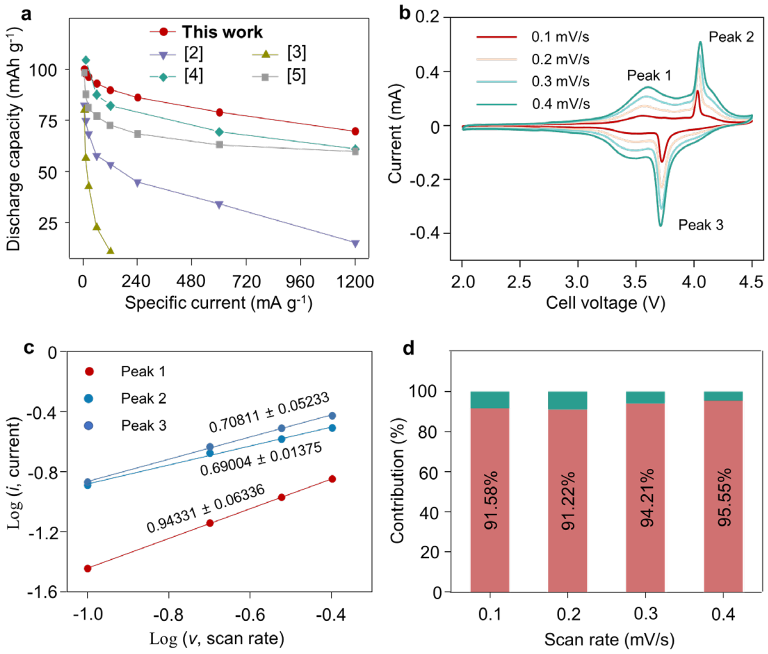

Supplementary Figure 22 | The kinetics investigation of NFS-H. a Comparison in rate performance of Na||NFS-H coin cells with reported literatures.[2–5] **b** CV curves at different scan rates of Na||NFS-H coin cells. **c** Log(i)-log(v) plot, and calculated b values with the errors. Error bars represent standard deviations for fitted curves. **d** Non-diffusion controlled contribution at various scan rates. The orange and dark green areas of the bars indicate the non-diffusion controlled (rapid redox process and electric double layer capacitance) and diffusion controlled contributions, respectively. Related discussion is presented in Supplementary Note 5.

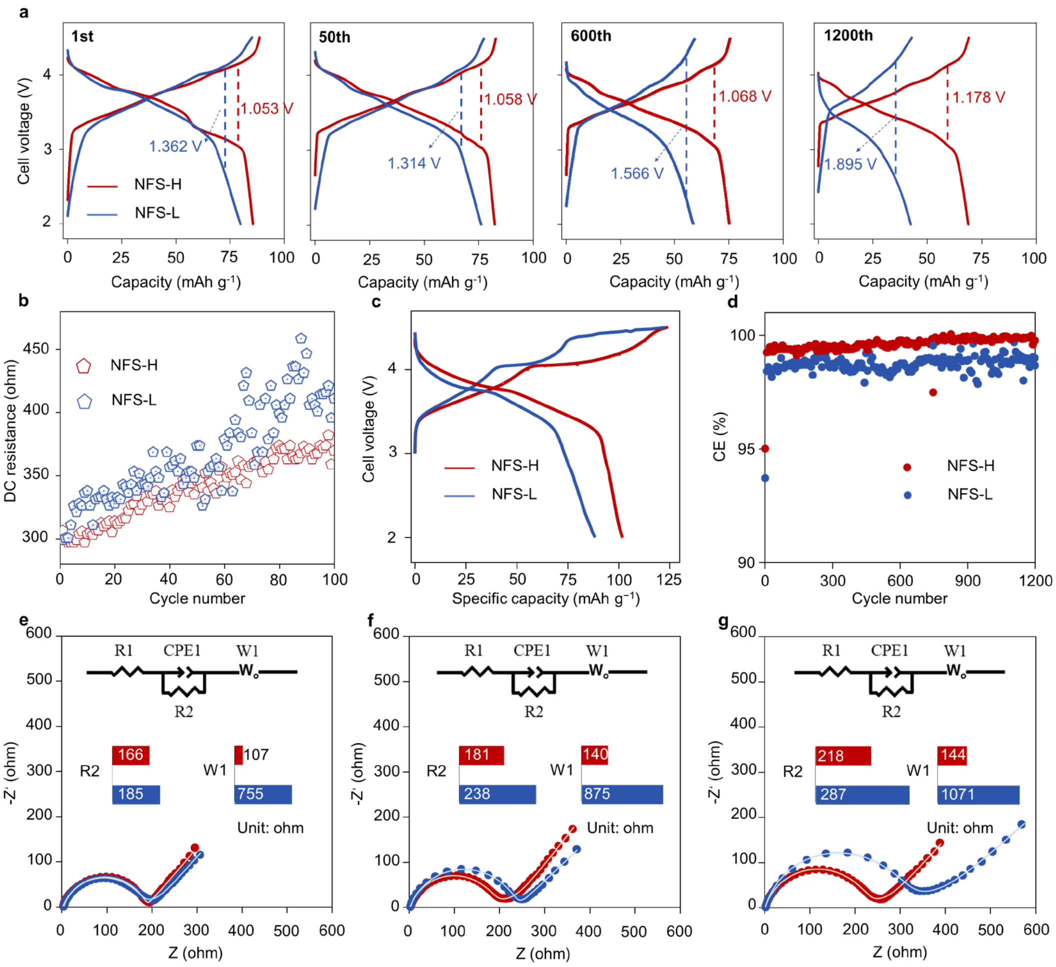

Supplementary Figure 23 | Electrochemical energy storage performance of Na||NFS-H- and Na||NFS-L coin cells at various cycles. a Charge-discharge curves at 1st, 50th, 600th, 1200th cycles at 60 mA g^{-1}. The vertical lines indicate the hysteresis of batteries at 95% discharged capacity (SOC%=5%). **b** DC internal resistance at 60 mA g^{-1}. **c** Initial charge-discharge curves at 6 mA g^{-1}. **d** CE performance. EIS plots after **e** initial cycle, **f** 20 cycles and (g) 50 cycles (line: fitted data; dot: pristine data). Related discussion is presented in Supplementary Note 6. In the fitted equivalent circuit, R1 represents the ohmic resistance in the cell system. Meanwhile, R2 and CPE1 (constant phase angle element) correspond to the resistance and capacitance of interfacial CEI. In addition, W1 represents the diffusion impedance. The unit of measurement for the numerical values of R and W is ohm.

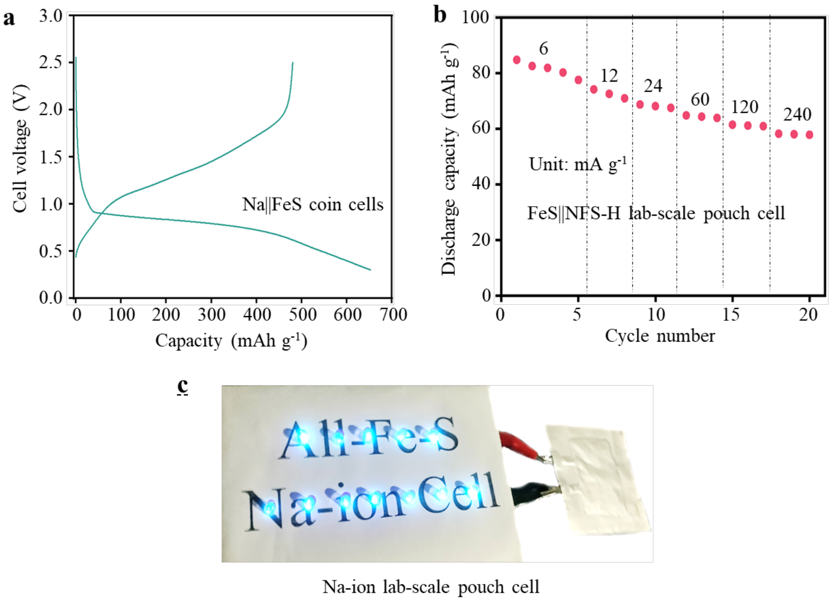

Supplementary Figure 24 | Electrochemical characterizations of FeS-based electrodes. **a** Charge-discharge profiles of Na||FeS coin cells at 100 mA g^{-1}. **b** Rate performance of FeS||NFS-H pouch cells. **c** The photographic picture of the FeS||NFS-H lab-scale pouch cell powering the led.

Supplementary table 1 | Atomic parameters of $Na_{2.26}Fe_{1.87}(SO_4)_3$ refined from Synchrotron high-pressure XRD Rietveld refinement of NFS-H. Space group: C2/c, *a*=12.7221 Å, *b*=12.8437 Å, *c*=6.5588 Å, *V*=966.879 Å^3.

Atom	x	y	z	Occ.	Iso.
Na1	0.500000	0.731537	0.750000	1.0	0.01478
Na2	0.000000	0.000000	0.000000	0.7430	0.03475
Na3	0.500000	0.982422	0.250000	0.5680	0.00460
Fe	0.730724	0.157770	0.147689	0.9359	0.00409
S1	0.000000	0.778764	0.750000	1.0	-0.00509
S2	0.760692	0.599142	0.866545	1.0	-0.00730
O1	0.070117	0.847020	0.710371	1.0	0.02388
O2	0.447019	0.209871	0.545086	1.0	-0.01689
O3	0.766531	0.665477	0.691271	1.0	-0.00203
O4	0.330900	0.998025	0.394337	1.0	0.02520
O5	0.362917	0.583915	0.680470	1.0	0.01652
O6	0.328971	0.150410	0.076752	1.0	0.01725

Supplementary table 2 | Atomic parameters of $Na_6Fe(SO_4)_4$ refined from Synchrotron high-pressure powder XRD Rietveld refinement of NFS-H. Space group: P 21/c, *a*=9.7018 Å, *b*=9.2715 Å, *c*=8.2570 Å, *V*=681.755 Å^3.

Atom	x	y	z	Occ.	Iso.
Na1	0.266766	0.007476	0.475326	1.0	-0.04763
Na2	0.437112	0.056891	0.081912	1.0	-0.04681
Na3	0.865898	0.118326	0.353204	1.0	-0.06723
Fe	0.000000	0.000000	0.000000	1.0	-0.05444
S1	0.140301	0.285214	0.252767	1.0	-0.01857
S2	0.644954	0.397857	0.306339	1.0	-0.04014
O1	0.105016	0.294197	0.184144	1.0	-0.08120
O2	0.129494	0.410082	0.086854	1.0	-0.09000
O3	0.143515	0.336069	0.265634	1.0	-0.09000
O4	0.188109	0.264697	0.246820	1.0	-0.09000
O5	0.722423	0.222255	0.312435	1.0	-0.07228
O6	0.606748	0.500504	0.466667	1.0	-0.09000
O7	0.796952	0.432058	0.355587	1.0	-0.09000
O8	0.647600	0.330134	0.364212	1.0	-0.09000

Supplementary table 3 | Atomic parameters of $Na_{2.26}Fe_{1.87}(SO_4)_3$ refined from X-ray diffraction Rietveld refinement of NFS-H. Space group: C2/c, *a*=12.645 Å, *b*=12.7659 Å, *c*=6.5158 Å, *V*=948.851314 Å^3.

Atom	x	y	z	Occ.	Iso.
Na1	0.500000	0.726600	0.750000	1.0	-0.090000
Na2	0.000000	0.000000	0.000000	0.7130	0.009870
Na3	0.500000	0.957000	0.250000	0.5432	0.103120
Fe	0.732320	0.157200	0.136200	0.9359	-0.002520
S1	0.000000	0.782100	0.750000	1.0	-0.053870
S2	0.746900	0.590900	0.871000	1.0	-0.044890
O1	0.097000	0.831000	0.703000	1.0	-0.077720
O2	0.456000	0.209000	0.553000	1.0	-0.077720
O3	0.747100	0.651000	0.677000	1.0	0.405550
O4	0.354500	1.001400	0.376000	1.0	-0.084500
O5	0.402000	0.570800	0.676000	1.0	-0.028500
O6	0.317000	0.146800	0.037000	1.0	-0.076670

Supplementary table 4 | Atomic parameters of $Na_6Fe(SO_4)_4$ refined from X-ray diffraction Rietveld refinement of NFS-H. Space group: P 21/c, *a*=9.797 Å, *b*=9.217 Å, *c*=8.199 Å, *V*=678.955615 Å^3.

Atom	x	y	z	Occ.	Iso.
Na1	0.310800	0.015500	0.467600	1.0	0.015070
Na2	0.445300	0.151600	0.091000	1.0	0.025580
Na3	0.889100	0.138200	0.316400	1.0	0.019880
Fe	0.000000	0.000000	0.000000	1.0	0.010890
S1	0.140000	0.305300	0.224600	1.0	0.006330
S2	0.661500	0.352900	0.347100	1.0	0.007350
O1	0.030900	0.184500	0.154600	1.0	0.015320
O2	0.136600	0.397200	0.076200	1.0	0.015580
O3	0.095600	0.393800	0.343300	1.0	0.017480
O4	0.288700	0.246100	0.320100	1.0	0.020640
O5	0.669200	0.192600	0.360100	1.0	0.018870
O6	0.647900	0.415000	0.502600	1.0	0.019120
O7	0.801100	0.406300	0.333800	1.0	0.015700
O8	0.537600	0.397100	0.183000	1.0	0.017730

Supplementary table 5 | Impedance parameters of the fitted equivalent circuit of Na||NFS-H coin cells after initial cycle (at discharge 2.0 V) at various temperatures. Related figure is shown in Supplementary Figure 19.

Temperatures	R1 / ohm	R2 / ohm	R3 / ohm	W1-R / ohm
298 K	3.729	6.358	327.8	614.9
308 K	2.702	12.06	149.1	122.6
318 K	2.808	17.12	65.86	88.13
328 K	2.489	28.74	22.01	63.41

Supplementary table 6 | The comparison of Na^+ diffusion coefficient of NFS-H cathode with various recently reported analogues and NASICON-type cathodes in SiBs.

Positive electrode	Negative electrode	Electrolyte solution	Voltage range (V)	Na^+ diffusion coefficient ($cm^2\ s^{-1}$)	Ref.
**NFS-H**	**Na metal**	**1M $NaClO_4$ EC/PC (1:1, vol) with 5% FEC**	**2.0–4.5 V**	**$10^{-12.44}$ – $10^{-10.52}$**	**This work**
$Na_2Fe(SO_4)_2$/C	Na metal	/	1.5–4.2 V	$10^{-11.76}$ (Charged 4.2 V)	[1]
$Na_2Fe(SO_4)_2$@rGO/C	Na metal	1M $NaClO_4$ EC/PC (1:1, vol) with 5% FEC	2.0–4.5 V	$10^{-11.92}$ (Charged 4.5 V)	[2]
$Na_{2.85}Fe_{1.57}(SO_4)_3$@N-rGO	Na metal	1M $NaClO_4$ EC/PC (1:1, vol)	2.0–4.5 V	$10^{-12.9}$ (Charged 4.5 V)	[4]
$Na_2FeM(SO_4)_3$	Na metal	1M $NaPF_6$ EC/DEC (1:1, vol)	2.0–4.5 V	10^{-14} – 10^{-11}	[6]
$Na_{2.4}Fe_{1.8}(SO_4)_3$	Na metal	1M $NaClO_4$ PC with 10% FEC	2.0–4.5 V	$10^{-11.84}$ – $10^{-10.63}$	[7]
$Na_6Fe_5(SO_4)_8$/CNT	Na metal	1M $NaClO_4$ PC	2.0–4.5 V	$10^{-12.69}$ – $10^{-12.2}$	[8]
$Na_{2+2x}Fe_{2-x}(SO_4)_3$@rGO	Na metal	1M $NaClO_4$ EC/PC (1:1, vol)	2.0–4.5 V	$10^{-11.42}$ – $10^{-11.34}$	[9]
$Na_2Fe_2(SO_4)_3$@C@GO	Na metal	1M $NaClO_4$ EC/PC (1:1, vol) with 5% FEC	3.2–4.1V	10^{-12} – $10^{-10.8}$	[10]
$Na_{2+2x}Fe_{2-x}(SO_4)_3$@graphene	Na metal	1M $NaClO_4$ EC/PC (1:1, vol) with 2% FEC	2.75–4.3 V	$10^{-13.79}$ (Charged 4.3 V)	[11]
$Na_{2.45}Fe_{1.775}(SO_4)_3$/rGO	Na metal	1M $NaClO_4$ EC/PC (1:1, vol) with 5% FEC	3.0–4.2 V	$10^{-11.05}$ – $10^{-10.53}$	[12]
$Na_2Fe(SO_4)_2\cdot 2H_2O$/C	Na metal	1M $NaClO_4$ PC	1.5–4.0 V	10^{-16} – 10^{-11}	[13]
$Na_{2+2x}Fe_{2-x}(SO_4)_3$@ PCNF	Na metal	1M $NaClO_4$ PC	2.0–4.5 V	$10^{-13.9}$ – $10^{-12.2}$	[14]
$Na_{2+2x}Fe_{2-x}(SO_4)_3$/SWNT	Na metal	1M $NaClO_4$ PC	2.0–4.5 V	$10^{-12.33}$	[15]

$Na_3V_2(PO_4)_2F_3$@RuO$_2$	Na metal	1M $NaClO_4$ EC/DMC (1:1, vol) with 5% FEC	2.5–4.3 V	$10^{-12.57}$ (Charged 4.1 V)	[16]
$Na_3V_2(PO_4)_2F_3$	Na metal	1M $NaClO_4$ EC/PC (1:1, vol) with 10% FEC	2.0–4.4 V	$10^{-11.10}$ – $10^{-9.77}$	[17]
$Na_3V_2(PO_4)_3$	Na metal	1M $NaClO_4$ EC/PC (1:1, vol) with 5% FEC	2.3–4.3 V	$10^{-9.88}$ – $10^{-9.04}$	[18]
$NaVPO_4F$/C	Na metal	1M $NaPF_6$ EC/DMC (1:2, vol) with 0.7 mmol FEC	2.5–4.0 V	$10^{-10.59}$ – $10^{-10.51}$	[19]
$Na_3V_2(PO_4)_2F_3$⊂GN	Na metal	1M $NaClO_4$ PC with 5% FEC	2.3–4.3 V	$10^{-10.01}$ – $10^{-9.74}$	[20]
$Na_3V_2(PO_4)_2F_3$@rGO	Na metal	1M $NaClO_4$ EC/DMC (1:1, vol) with 5% FEC	2.0–4.3 V	$10^{-10.35}$ – $10^{-9.60}$	[21]

Supplementary table 7 | The comparison of cycling performance of NFS-H cathode with various recently reported analogues in SiBs.

Positive electrode	Positive electrode's mass loading (mg cm^{-2})	Negative electrode	Reversible capacity (mAh g^{-1})	Capacity retention	Ref.
**NFS-H**	**1.5–2.0**	**Na metal**	**102 (6 mA g^{-1})**	**80.7% (1300 cycles, 60 mA g^{-1})**<br>**65.3% (5700 cycles, 600 mA g^{-1})**	**This work**
$Na_2Fe(SO_4)_2$/C	2.0	Na metal	82 (12 mA g^{-1})	60% (100 cycles, 24 mA g^{-1})	[1]
$Na_2Fe(SO_4)_2$@rGO/C	1.5	Na metal	85 (6 mA g^{-1})	81.2% (200 cycles, 60 mA g^{-1})	[2]
$Na_{2.85}Fe_{1.57}(SO_4)_3$@N-rGO	/	Na metal	93.2 (6 mA g^{-1})	90% (200 cycles, 24 mA g^{-1})<br>87% (200 cycles, 120 mA g^{-1})<br>86% (300 cycles, 600 mA g^{-1})<br>83% (400 cycles, 1200 mA g^{-1})	[4]
$Na_{2.4}Fe_{1.8}(SO_4)_3$	1.2–1.5	Na metal	80 (6 mA g^{-1})	85% (50 cycles, 6 mA g^{-1})	[9]
$Na_2Fe_2(SO_4)_3$@C@GO	1.6	Na metal	108 (12 mA g^{-1})	90% (300 cycles, 24 mA g^{-1})	[10]
$Na_{2+2x}Fe_{2-x}(SO_4)_3$@graphene	/	Na metal	106 (12 mA g^{-1})	85% (200 cycles, 120 mA g^{-1})	[11]
$Na_{2.45}Fe_{1.775}(SO_4)_3$	2.0	Na metal	90 (12 mA g^{-1})	89% (100 cycles, 12 mA g^{-1})<br>84% (500 cycles, 2400 mA g^{-1})	[12]
$Na_{2+2x}Fe_{2-x}(SO_4)_3$@rGO	5.0	Na metal	98.6 (12 mA g^{-1})	95.7% (100 cycles, 60 mA g^{-1})<br>90.8% (300 cycles, 600 mA g^{-1})<br>85.9% (500 cycles, 1200 mA g^{-1})<br>80.8% (2000 cycles, 3600 mA g^{-1})<br>73.3% (2000 cycles, 7200 mA g^{-1})	[22]
$Na_2Fe_2(SO_4)_3$	1.0	Na metal	102 (6 mA g^{-1})	/	[23]

Supplementary table 8 | Comprehensive evaluation on various sodium-ion battery systems.

Battery systems	Specific energy (Wh kg^{-1})	Element abundance	Electrode costs	Recyclability	Ref.
**$FeS||Na_{2.26}Fe_{1.87}(SO_4)_3$**	**168.2**	**Good**	**Good**	**Good**	**This work**
$HC||Na_2FePO_4F$	135.8	Fair	Good	Fair	[24]
$NaTi_2(PO_4)_3||Na_{1.64}Ni[Fe(CN)_6]_{0.92}$ $1.83H_2O$	Poor	Fair	Fair	Poor	[25]
$HC||Na_{0.76}Cu_{0.2}2Fe_{0.30}Mn_{0.48}O_2$	177.4	Fair	Poor	Fair	[26]
$HC||Na_3(VOPO_4)_2F$	Good	Poor	Fair	Fair	[27]

Supplementary table 9 | Calculated cost parameters of various electrode materials.

Materials	Main raw materials	Price (￥)/ Quantity (g)	Heat-treatment process	Ref.
**NFS-H**	**1. Sodium sulfate anhydrous**<br>**2. Iron sulfate heptahydrate**<br>**3. Citric acid monohydrate**	**30/500**<br>**34/500**<br>**37/500**	**Low-temperature calcination (350 °C)**	**This work**
$Na_{1.64}Ni[Fe(CN)_6]_{0.92}$ $1.83H_2O$	1. Sodium hexacyanoferrate (II)<br>2. Nickel chloride hexahydrate<br>3. Sodium citrate<br>4. Sodium chloride<br>5. Polyvinylpyrrolidone	98/500<br>117/500<br>94/500<br>31/500<br>215/500	Low-temperature drying (120 °C)	[25]
Na_2FePO_4F@C	1. Citric acid monohydrate<br>2. Polyvinylpyrrolidone<br>3. Iron(II) acetate<br>4. Sodium dihydngen phoshate anhydrous<br>5. Sodium fluoride	37/500<br>215/500<br>1999/100<br>119/500<br>48/500	High-temperature calcination (600 °C)	[24]
$Na_{0.76}Cu_{0.2}2Fe_{0.30}Mn_{0.48}O_2$	1. Polyvinylpyrrolidone<br>2. Acetate<br>3. Manganese acetate tetrahydrate<br>4. Sodium nitrate<br>5. Copper nitrate trihydrate<br>6. Iron nitrate nonahydrate	215/500<br>369/2500 mL<br>69/500<br>125/500<br>98/500<br>411/500	High-temperature calcination (850 °C)	[26]
$Na_3(VOPO_4)_2F$	1. Sodium metavanadate<br>2. Sodium phosphate monobasic dihydrate<br>3. Sodium fluoride<br>4. Hydroxylammonium chloride	299/250<br>49/500<br>48/500<br>94/500	Low-temperature drying (120 °C)	[27]

Supplementary Note 1

In Supplementary Fig. 8, to verify the effect of calculation platform on Na-ion diffusion, we tried softBV software to calculate the ion migration barrier of $Na_{2.26}Fe_{1.87}(SO_4)_3$ and $Na_6Fe(SO_4)_4$ crystals in this work. Migration pathways are analyzed as regions of low $E_{BVSE(Na)}$ in grids spanning the structure model with a resolution of ca. 0.1 Å^3. Visibly, the $Na_{2.26}Fe_{1.87}(SO_4)_3$ crystal provides intermittent and narrow channels for Na^+ migration along the b-axis and c-axis direction across a zigzag path between two equivalent positions, while the $Na_6Fe(SO_4)_4$ crystal possesses successive and broad Na^+ migration pathways along the 3D (a, b, c-axis) directions, which is consistent with the result obtained from high-throughput computational platform for battery materials.

Further calculations on energy barrier reveal that Na^+ migrates mainly along 1D and 2D directions for both $Na_{2.26}Fe_{1.87}(SO_4)_3$ and $Na_6Fe(SO_4)_4$ crystals. Differently, compared with the result obtained from high-throughput computational platform for battery materials, the calculated values of Na^+ transfer barrier via SoftBV software are lower, which is closer to the values reported in the literature [23,28]. The $Na_{2.26}Fe_{1.87}(SO_4)_3$ crystal show the Na^+ transfer barrier of 0.891 eV (along 1D paths), 1.437 eV (along 2D paths) and 999 eV (along 3D paths), respectively. While the $Na_6Fe(SO_4)_4$ crystal show the Na^+ transfer barrier of 0.542 eV (along 1D paths), 0.751 eV (along 2D paths) and 1.659 eV (along 3D paths), respectively. A lower barrier for Na^+ migration within Na1-Na2 pathways also supports the superionic conductor property of $Na_6Fe(SO_4)_4$. Therefore, it is reasonably concluded that different calculation platform should take responsibility for the difference on energy barriers of Na^+ migration between this work and reported literature.

Supplementary Note 2

In Supplementary Fig. 9, X-ray diffraction pattern (XRD) demonstrates the successful synthesis of $Na_6Fe(SO_4)_4$ phase materials. Furthermore, trace amount of $NaSO_4$ and Na_5FeO_4 materials are also included in the analysis as an impurity, which may originate from the inexhaustive solid reaction and high-temperature oxidation during synthesis process. Galvanostatic charge/discharge (GCD) curves shows its poor sodium storage ability with a low specific capacity of 10 mAh g^{-1}, which is consistent with the report by Jiang et al.[1]

In the fitted equivalent circuit, R1 represents the ohmic resistance in the cell system. Meanwhile, R2 and CPE1 (constant phase angle element) are corresponded to the resistance and capacitance of interfacial CEI. In addition, R3, CPE2 and W1 represent the faradaic impedance of the reaction, the interphase double layer capacitance and the diffusion impedance, respectively. Based on the Fick's laws (equation 2), the Na^+ diffusion coefficient inside the as-synthesized $Na_6Fe(SO_4)_4$ electrode was calculated to be $10^{-9.59}$ cm^2 s^{-1}.

$$D = \frac{R^2T^2}{2A^2n^4F^4C^2\sigma^2} \qquad \text{(Equation 2)}$$

R is the gas constant, T represents the temperature, A is the area of the electrode, F is the Faraday constant, n is the number of electrons per molecule in the charge-discharge reaction and σ is the slope of the $Z'–\omega^{-1/2}$ curve.

Based on equation 1 in Methods, the diffusion coefficients of Na^+ ions at different voltage states were also calculated. The diffusion coefficients range from $10^{-10.543}$ to $10^{-7.213}$ cm^2 s^{-1} within the voltage window of 2.0–4.5 V, which are higher than those of the alluaudite-type $Na_{2+2x}Fe_{2-x}(SO_4)_3$. For instance, Chou's group reported that the alluaudite-type $Na_2Fe_2(SO_4)_3$ materials without visible $Na_6Fe(SO_4)_3$ impurity featured a diffusion coefficients range from 10^{-12} to $10^{-10.8}$ cm^2 s^{-1}.[10] Thus, an improved Na^+ conductivity can be determined in $Na_6Fe(SO_4)_4$ phase, which supports our theoretical calculation and electrochemical results.

Supplementary Note 3

In Supplementary Fig. 11, the exposed crystal planes of NFS-H materials were further confirmed via HAADF-STEM images at various regions. The result indicates the rich distribution of (11-2) and (240) crystal planes of NFS-H particle. Except them, other crystal planes like (13-1), (31-3) and (200) are also observed. This is highly consistent with the information shown by XRD results. As we known, the information of crystal planes obtained via XRD include many particles from surface to body. Thus, it can be reasonably inferred that in NFS-H materials, not all exposed crystal planes exist in (11-2) structure. But, it is the most exposed one. Furthermore, compared with $Na_{2+2x}Fe_{2-x}(SO_4)_3$ reported in literatures,[1,7,8] NFS-H shows a relatively stronger (11-2) peak.

Supplementary Note 4

In Supplementary Fig. 21, the galvanostatic charge-discharge test at 6 mA g^{-1} of Na||NFS-H cell shows a reversible capacity reaches 101.3 mAh g^{-1} and an average cell discharge voltage of 3.75 V at initial cycle. In contrast, the Na||NFS-L cell with less ionic-conducting phase features a lower capacity (89.7 mAh g^{-1}) and average cell discharge voltage of 3.66 V. In addition, benefited from the good ionic conducting of $Na_6Fe(SO_4)_4$, the NFS-H-containing cell shows a lower hysteresis of 1.098 V than that (1.541 V) of NFS-L-containing cell. It indicates the improved reaction kinetics of ionic-conducting $Na_6Fe(SO_4)_4$ phase, facilitating the decreased electrochemical polarization in NFS-H. At high currents, NFS-H cathode shows a voltage inflexion around 3.0 V in discharge curves. According to the report by Jiang et al., it may be attributed to the Fe^{2+}/Fe^{3+} transformation in pure $Na_6Fe(SO_4)_4$@C cathode.[1] With the current increases, the electrochemistry belonging to $Na_6Fe(SO_4)_4$ phase is more relevant, indicating the possible key role of $Na_6Fe(SO_4)_4$ phase in reaction kinetics at high currents. While NFS-L does not show voltage inflexion at high currents (600 and 1200 mA g^{-1}). In order to show this more clearly, we have gathered the relevant data graph from the reported literature. Although pure $Na_6Fe(SO_4)_4$@C cathode shows poor sodium storage activity and capacity, a discernable voltage inflexion occurs in its discharge curves around 3.0 V.[1] Moreover, peaks located at 2.89/3.05 V and 2.62/2.83 V in CV curves of Na||NFS-H and Na||NFS-L cells may be an electrochemical characteristic of Fe^{2+}/Fe^{3+} redox of $Na_6Fe(SO_4)_4$ phase in NFS-H.[2]

The GCD curves for both NFS-H and NFS-L show two plateau-like features (with an abrupt change at ~4.0 V). The sloping voltage curve over the entire range of Na composition suggests a single-phase homogeneous reaction mechanism involving minimal volume change. According to the result reported by Atsuo Yamada., throughout the whole initial charging process of alluaudite-type $Na_{2+2x}Fe_{2-x}(SO_4)_3$ compounds, Na extraction occurs primarily at Na3 followed by the Na1 and Na2 sites.[29] Especially, the broad peak observed at 3.67 V corresponds to Na extraction primarily occurred at the Na3 site with a small migration energy between Na3−Na3 sites. While the bulge at the voltage curve at 3.9 V is attributed to the localized lattice distortion due to the inherent reflection for the Fe migration into vacant Na(1) sites.[10] On the sharp peak in the CV curve at 4.06 V in the initial charge process, it is associated with Na extraction from Na1 site, which then starts to induce Fe^{3+} migration from the Fe site into the vacant Na1 site.

Supplementary Note 5

In Supplementary Fig. 22, the sodium storage kinetics of Na||NFS-H cell is also investigated by CV curves at the scan rates of 0.1−0.4 mV s^{-1}. With the increased scan rate, anodic peaks shift slightly to lower potentials and cathodic peaks are the opposite, but all CV curves display similar shapes. According to the relationship between scan rate (v) and peak current (*i*), $i = av^b$, the b values for the peak 1, 2 and 3 are0.988, 0.633 and 0.734, respectively, which are within desirable range, suggesting a non-diffusion-controlled diffusion process. The quantification of non-diffusion controlled capacitive contribution is further computed by the following equation, $i = k_1v + k_2v^{1/2}$, which increases with the larger scan rate, up to 95.55% at 0.4 mV s^{-1}. The reason for high non-diffusion-controlled charge contribution may include two points: (1) The obtained NFS-H material feature micron-sized polyhedra consisting of massively stacked nanoparticles. The nanoscale size of active material in NFS-H leads to short diffusion lengths for Na ions in electrodes (even negligible ion diffusion, approaching a surface process). (2) The introduction of minor ionic-conductive $Na_6Fe(SO_4)_4$ phase improves the ionic kinetics of NFS-H inside particle bulk, greatly accelerating the redox kinetics during electrochemical process, which prevents it from being a major barrier for Na^+ diffusion at full scale in batteries.

Supplementary Note 6

In Supplementary Fig. 23, during long-term cycling at 60 mA g^{-1}, Na||NFS-H and Na||NFS-L cells show increased potential differences and electrochemical polarization, accompanied with decreased capacity and voltage output. Furthermore, EIS measurements during the initial 50 cycles show continuously increased interfacial resistances (R2) and diffusion impedance (W1) for Na||NFS-H and Na||NFS-L cells. Thus, the capacity fading of NFS-H and NFS-L cathodes during long-term cycles is mainly from the increased electrochemical polarization. Detailed reasons might include two points:

(1) Interfacial evolution. Upon the initial charging process, the electrolyte is oxidized on cathode surfaces to form a protective CEI layer. And a lower initial coulombic efficiency (72.2%) of Na||NFS-L cell indicates a more severe decomposition of electrolytes, which might induce the formation of a thicker CEI and bring greater barrier for ion transfer across the interface. In subsequent cycles, side reactions may continuously occur at cathode/electrolyte interface, accompanied by the thickening of CEI and increased interfacial resistance. For example, after 70 cycles at 60 mA g^{-1}, the thickness of CEI formed on NFS-H increases to 12–17 nm from 10–14 nm at 1st cycle (Supplementary Fig. 18). And the interfacial resistance (R2) increases by 31.3% and 55.1% for NFS-H and NFS-L during the initial 50 cycles, respectively.

(2) The fading of heterostructure within bulk. During repeated cycles, the insertion/extraction of Na^+ leads to changes in the molar volume of $Na_{2.26}Fe_{1.87}(SO_4)_3$ and $Na_6Fe(SO_4)_4$ phases, which may induce mechanical stress and strain to their crystals. Thus, accidental disconnection between two phases is possible in local regions, which might damage the ion-conduction networks and boost the energy barrier of Na^+ migration inside the electrodes. EIS measurements also reveal increased diffusion impedance by 34.6% and 41.9% for NFS-H and NFS-L during the initial 50 cycles, respectively. Moreover, due to insufficient ion diffusion channels, sluggish reaction kinetics of NFS-L might maintain from initial cycle and induce continuous reaction irreversibility.

Therefore, during long-time cycle, the interface resistance caused by electrolyte's decomposition and the diffusion resistance within the bulk accumulate continuously, resulting in increased potential difference and decreased capacity output of the electrodes.

Although continued electrochemical fading occurs, improvement in electrochemical durability is still achieved in Na||NFS-H cell due to richer ionic-conducting $Na_6Fe(SO_4)_4$ phase. During 1200 cycles at 60 mA g^{-1}, Na||NFS-H cell achieves a higher average coulombic efficiency of 99.59% than that (98.66%) of the Na||NFS-L cell, which may be attributed to the enhanced reaction reversibility and kinetics. For instance, compared to the NFS-L, the NFS-H shows a lower potential difference (1.603 V vs. 2.203 V at 1200th cycle) and an increase of diffusion impedance (34.6% vs. 41.9% after 50 cycles), indicating its good maintaining of electrochemical polarization. In addition, for the Na||NFS-L cell after the cycles (1st, 20th and 50th), the diffusion impedance (W1) is larger than interfacial resistance (R2) in value, indicating the ions diffusion inside the bulk of electrodes contributes the major impedance of batteries. However, the case did not occur in Na||NFS-H cell. Therefore, it is reasonably inferred that, with the increase of introduced ionic-conducting $Na_6Fe(SO_4)_4$ phase, sufficient ion diffusion

channels could be effectively built in electrodes. It facilitates the good maintenance of fast reaction kinetics, ensuring good reaction reversibility and capacity maintenance of electrodes.

Supplementary reference

1. Pan, W. *et al.* $Na_2Fe(SO_4)_2$: an anhydrous 3.6 V, low-cost and good-safety cathode for a rechargeable sodium-ion battery. *J. Mater. Chem. A* **7**, 13197–13204 (2019).
2. Yao, G. *et al.* Facile synthesis of hierarchical $Na_2Fe(SO_4)_2$@rGO/C as high-voltage cathode for energy density-enhanced sodium-ion batteries. *J. Energy Chem.* **50**, 387–394 (2020).
3. Jungers, T., Mahmoud, A., Malherbe, C., Boschini, F. & Vertruyen, B. Sodium iron sulfate alluaudite solid solution for Na-ion batteries: Moving towards stoichiometric $Na_2Fe_2(SO_4)_3$. *J. Mater. Chem. A* **7**, 8226–8233 (2019).
4. Wang, W. *et al.* A high voltage cathode of $Na_{2+2x}Fe_{2-x}(SO_4)_3$ intensively protected by nitrogen-doped graphene with improved electrochemical performance of sodium storage. *J. Mater. Chem. A* **6**, 4354–4364 (2018).
5. Fang, Y. *et al.* An advanced low-cost cathode composed of graphene-coated $Na_{2.4}Fe_{1.8}(SO_4)_3$ nanograins in a 3D graphene network for ultra-stable sodium storage. *J. Energy Chem.* **54**, 564–570 (2021).
6. Plewa, A. *et al.* Facile aqueous synthesis of high performance $Na_2FeM(SO_4)_3$ (M = Fe, Mn, Ni) alluaudites for low cost Na-ion batteries. *J. Mater. Chem. A* **8**, 2728–2740 (2020).
7. Hou, J., Wang, W., Feng, P., Wang, K. & Jiang, K. A surface chemistry assistant strategy to high power/energy density and cost-effective cathode for sodium ion battery. *J. Power Sources* **453**, 227879 (2020).
8. Li, S. *et al.* A nanoarchitectured $Na_6Fe_5(SO_4)_8$/CNTs cathode for building a low-cost 3.6 V sodium-ion full battery with superior sodium storage. *J. Mater. Chem. A* **7**, 14656–14669 (2019).
9. Liu, X., Tang, L., Xu, Q., Liu, H. & Wang, Y. G. Ultrafast and ultrastable high voltage cathode of $Na_{2+2x}Fe_{2-x}(SO_4)_3$ microsphere scaffolded by graphene for sodium ion batteries. *Electrochim. Acta* **296**, 345–354 (2019).
10. Chen, M. *et al.* A novel graphene oxide wrapped $Na_2Fe_2(SO_4)_3$/C cathode composite for long life and high energy density sodium - ion batteries. *Adv. Energy Mater.* **8**, 1800944 (2018).
11. Liu, Y. *et al.* Insight into the multirole of graphene in preparation of high performance $Na_{2+2x}Fe_{2-x}(SO_4)_3$ cathodes. *ACS Sustain. Chem. Eng.* **6**, 16105–16112 (2018).
12. Zhang, M. *et al.* Reduced graphene oxide wrapped alluaudite $Na_{2+2x}Fe_{2-x}(SO_4)_3$ with high rate sodium ion storage properties. *J. Alloys Compd.* **752**, 267–273 (2018).
13. Meng, Y., Li, Q., Yu, T., Zhang, S. & Deng, C. Architecture-property relationships of zero-, one- and two-dimensional carbon matrix incorporated $Na_2Fe(SO_4)_2 \cdot 2H_2O$/C. *CrystEngComm* **18**, 1645–1654 (2016).
14. Yu, T. *et al.* First exploration of freestanding and flexible $Na_{2+2x}Fe_{2-x}(SO_4)_3$@porous carbon nanofiber hybrid films with superior sodium intercalation for sodium ion batteries. *Phys. Chem. Chem. Phys.* **18**, 26933–26941 (2016).
15. Meng, Y., Yu, T., Zhang, S. & Deng, C. Top-down synthesis of muscle-inspired alluaudite $Na_{2+2x}Fe_{2-x}(SO_4)_3$/SWNT spindle as a high-rate and high-potential cathode for sodium-ion batteries. *J. Mater. Chem. A* **4**, 1624–1631 (2016).

16. Zhang, Y., Xun, J., Zhang, K., Zhang, B. & Xu, H. 2D-lamellar stacked $Na_3V_2(PO_4)_2F_3$@RuO_2 as a high-voltage, high-rate capability and long-term cycling cathode material for sodium ion batteries. *J. Mater. Chem. A* **10**, 11163–11171 (2022).
17. Subramanian, Y. *et al.* Optimizing high voltage $Na_3V_2(PO_4)_2F_3$ cathode for achieving high rate sodium-ion batteries with long cycle life. *Chem. Eng. J.* **403**, 126291 (2021).
18. Yang, Z. *et al.* High performance cathode material based on $Na_3V_2(PO_4)_2F_3$ and $Na_3V_2(PO_4)_3$ for sodium-ion batteries. *Energy Storage Mater.* **25**, 724–730 (2020).
19. Chen, C., Li, T., Tian, H., Zou, Y. & Sun, J. Building highly stable and industrial $NaVPO_4F$/C as bipolar electrodes for high-rate symmetric rechargeable sodium-ion full batteries. *J. Mater. Chem. A* **7**, 18451–18457 (2019).
20. Zhu, C. *et al.* A high power-high energy $Na_3V_2(PO_4)_2F_3$ sodium cathode: investigation of transport parameters, rational design and realization. *Chem. Mater.* **29**, 5207–5215 (2017).
21. Cai, Y. *et al.* Caging $Na_3V_2(PO_4)_2F_3$ microcubes in cross-linked graphene enabling ultrafast sodium storage and long-term cycling. *Adv. Sci.* **5**, 1800680 (2018).
22. Dwibedi, D. *et al.* Ionothermal synthesis of high-voltage alluaudite $Na_{2+2x}Fe_{2-x}(SO_4)_3$ sodium insertion compound: structural, electronic, and magnetic insights. *ACS Appl. Mater. Interfaces* **8**, 6982–6991 (2016).
23. Barpanda, P., Oyama, G., Nishimura, S., Chung, S.-C. & Yamada, A. A 3.8-V earth-abundant sodium battery electrode. *Nat. Commun.* **5**, 4358 (2014).
24. Wang, F. *et al.* Realizing a high‐performance Na‐storage cathode by tailoring ultrasmall Na_2FePO_4F nanoparticles with facilitated reaction kinetics. *Adv. Sci.* **6**, 1900649 (2019).
25. Peng, J. *et al.* Defect-free-induced Na^+ disordering in electrode materials. *Energy Environ. Sci.* **14**, 3130–3140 (2021).
26. Shen, Q. *et al.* Dual‐strategy of cation‐doping and nanoengineering enables fast and stable sodium‐ion storage in a novel Fe/Mn‐based layered oxide cathode. *Adv. Sci.* **7**, 2002199 (2020).
27. Shen, X. *et al.* Rapid mechanochemical synthesis of polyanionic cathode with improved electrochemical performance for Na-ion batteries. *Nat. Commun.* **12**, 2848 (2021).
28. Wong, L. L., Chen, H. M. & Adams, S. Sodium-ion diffusion mechanisms in the low cost high voltage cathode material $Na_{2+\delta}Fe_{2-\delta/2}(SO_4)_3$. *Phys. Chem. Chem. Phys.* **17**, 9186–9193 (2015).
29. Oyama, G. *et al.* Sodium intercalation mechanism of 3.8 V class alluaudite sodium iron sulfate. *Chem. Mater.* **28**, 5321–5328 (2016).
